# Altered Neuroanatomical Signatures of Patients With Treatment-Resistant Schizophrenia Compared to Patients With Early-Stage Schizophrenia and Healthy Controls

**DOI:** 10.3389/fpsyt.2022.802025

**Published:** 2022-05-18

**Authors:** Congcong Liu, Woo-Sung Kim, Jie Shen, Uyanga Tsogt, Nam-In Kang, Keon-Hak Lee, Young-Chul Chung

**Affiliations:** ^1^Department of Psychiatry, Medical School, Jeonbuk National University, Jeonju, South Korea; ^2^Research Institute of Clinical Medicine of Jeonbuk National University-Biomedical Research Institute of Jeonbuk National University Hospital, Jeonju, South Korea; ^3^Department of Psychiatry, Maeumsarang Hospital, Wanju, South Korea; ^4^Department of Psychiatry, Jeonbuk National University Hospital, Jeonju, South Korea

**Keywords:** cortical thickness, treatment-resistant schizophrenia, MRI, subcortical volume, verbal memory, early-stage schizophrenia

## Abstract

**Background:**

The relationship between brain structural changes and cognitive dysfunction in schizophrenia is strong. However, few studies have investigated both neuroanatomical abnormalities and cognitive dysfunction in treatment-resistant schizophrenia (TRS). We examined neuroanatomical markers and cognitive function between patients with TRS or early-stage schizophrenia (ES-S) and healthy controls (HCs). Relationships between neuroanatomical markers and cognitive function in the patient groups were also investigated.

**Methods:**

A total of 46 and 45 patients with TRS and ES-S and 61 HCs underwent structural magnetic resonance imaging (MRI) brain scanning and comprehensive cognitive tests. MRI scans were analyzed using the FreeSurfer to investigate differences in cortical surface area (CSA), cortical thickness (CT), cortical volume (CV), and subcortical volume (SCV) among the groups. Four cognitive domains (attention, verbal memory, executive function, and language) were assessed. Comparisons of neuroanatomical and cognitive function results among the three groups were performed.

**Results:**

A widespread reduction in CT was observed in patients with TRS compared to HCs, but differences in cortical thinning between TRS and ES-S patients were mainly limited to the inferior frontal gyrus and insula. Several subcortical structures (accumbens, amygdala, hippocampus, putamen, thalamus and ventricles) were significantly altered in TRS patients compared to both ES-S patients and HCs. Performance in the verbal memory domain was significantly worse in TRS patients compared to ES-S patients. A positive relationship between the thickness of the left middle temporal gyrus and the composite score for language was identified in patients with ES-S.

**Conclusions:**

Our findings suggest significant cognitive impairment and reductions in CT and SCV in individuals with TRS compared to those with ES-S and HCs. These abnormalities could act as biomarkers for earlier identification of TRS.

## Introduction

Previous research has shown that 20–30% of people with schizophrenia do not respond to treatment with antipsychotic medications (1), a condition known as treatment-resistant schizophrenia (TRS). The following consensus criteria for defining TRS were recently proposed: (1) for current symptoms, at least moderate severity with a duration of ≥12 weeks and at least moderate functional impairment and (2) for adequate treatment assessment of past responses, failure in at least two previous antipsychotic trials with a duration of ≥6 weeks at a therapeutic dosage equivalent to ≥600 mg of chlorpromazine per day (2). The social and economic burden of TRS is huge in that patients with TRS have high rates of smoking, alcohol abuse, substance abuse, and suicidal ideation. Annual costs are 3–11-fold higher in patients with TRS than those for general schizophrenia (3). This calls for urgent efforts to ensure the effective identification and treatment of such patients.

Research on neuroanatomical signatures of the brain is important for the identification and treatment of TRS. Previous studies on cortical thickness (CT) reported widespread reduction in patients with TRS compared to healthy controls (HCs) or additional reduction (dorso-lateral prefrontal cortex, or frontal, temporal and cingulate cortices) compared to non-treatment-resistant schizophrenia (NTRS) (4, 5). However, another study reported no difference between patients with TRS and controls at baseline (6). With respect to cortical volume (CV), several studies have demonstrated greater reductions in multiple regions in TRS (4, 7, 8). One systematic review of brain imaging studies of TRS indicated that the most-replicated finding was a greater reduction in gray matter in resistant patients, predominantly in frontal areas, compared with responsive patients (9). In terms of subcortical volume (SCV), reduced volumes of the globus pallidus, hippocampus, striatum, and thalamus have been reported in TRS (10, 11). However, conflicting results of comparisons have ranged from no difference to greater corpus callosum volume in TRS (12, 13). The inconsistent findings may be due to adopting different criteria for defining TRS causing heterogeneity of the participants. Hence, to resolve this issue, more research adopting the consensus criteria for TRS is needed. Another important consideration in TRS research is to choose an appropriate patient group to be compared. Most of previous studies used patients with NTRS as comparison group. Nevertheless, as we were more interested in preventing deteriorating progression from early-stage of illness to chronic resistant stage, patients with early-stage schizophrenia were chosen as comparison group.

Cognitive impairment, a core clinical feature of schizophrenia, is considered a strong predictor of long-term prognosis in patients (14). Some have proposed including cognitive impairment and global functioning in definitions of TRS (15, 16). Several studies investigating cognitive functioning in TRS reported overall cognitive impairment compared to NTRS, including poorer performance on tests of verbal abilities, memory, learning (17, 18), and attention (19) and lower processing speed, verbal fluency, cognitive flexibility, and executive function (20). In schizophrenia, subcortical structures (21) as well as cortical gray matter volume and CT (22) are associated with cognitive deficits. However, current evidence supporting the existence of relationships between neuroanatomical abnormalities and cognitive dysfunction in TRS is scarce. Previous studies reported an association between working memory deficits and lower hippocampal volume (10) and Stroop interference and myelin water fraction in the corpus callosum in TRS (23).

The aims of this study were to investigate neuroanatomical markers and cognitive dysfunction differentiating patients with TRS from those with early stage schizophrenia (ES-S) and HCs. In addition, we sought to explore the relationships between neuroanatomical abnormalities and cognitive function. We hypothesized that neuroanatomical abnormalities and cognitive dysfunction of patients with TRS and association of these two deficits would be more remarkable compared to patients with ES-S as well as HCs.

## Materials and Methods

### Participants

The study included 152 subjects comprising 46 patients with TRS, 45 patients with ES-S, and 61 HCs. The diagnosis of schizophrenia was based on the Diagnostic and Statistical Manual of Mental Disorders-5 (24), and each patient's diagnosis was decided on a consensus basis between the patient's physician and one of the study's authors. The exclusion criteria for patients were as follows: (a) alcohol or substance use disorder; (b) intellectual disability (IQ ≤70); (c) current or past neurological disease, serious medical illness, or pregnancy; and (d) claustrophobia. We matched the age, sex, and education level of two patient groups. Trained psychiatrists performed clinical evaluations using the Positive and Negative Syndrome Scale (PANSS) (25).

Treatment resistance was defined by the following criteria: (1) failure to respond to at least 6-week trials of at least two different antipsychotic medications administered in adequate doses (equivalent to at least 600 mg/day of chlorpromazine [CPZ]) and (2) persistence of clinically relevant positive or negative symptoms (at least one positive or negative symptom with a PANSS score of 4 or more) (26). We adopted these criteria from the minimum requirement, not the optimum requirement suggested by the treatment response and resistance in psychosis (TRRIP) working group consensus guidelines (2) and other guidelines (27). The second criterion was not applied to patients on clozapine because some of them did not have persistent positive or negative symptoms and were also considered as TRS in other study (28). The ES-S diagnoses included schizophrenia and schizophreniform disorder with an illness duration of 5 years or less. Antipsychotic doses at the time of assessment were standardized using the defined daily dose (DDD) following the guidelines available at http://www.whocc.no/atc_ddd_index (29). Among TRS patients, 26 were on clozapine alone (*n* = 2) or clozapine plus other antipsychotics and 20 were on no-clozapine in either single (*n* = 2) or combination. Among ES-S patients, 22 were antipsychotic-naïve (*n* = 12) or -free (*n* = 10). HCs were recruited through advertising and then interviewed using the non-patient version of the Structured Clinical Interview for DSM-IV (SCID-IV) (30). The exclusion criteria for HCs were the same as the patient group except that there should be no first-degree relative suffering from mental disorders. The age, sex, and education level of HCs were matched to those of patients. All patients were recruited at Jeonbuk National University Hospital and volunteered to participate in the study; all provided written informed consent. This study has been approved by the Ethics Committee of Jeonbuk National University Hospital (approval number: CUH 2012-08-001).

### Cognitive Test

Computerized neurocognitive tests (MaxMedica, Inc., Seoul, Korea) were administered to each subject within 1 month before a magnetic resonance imaging (MRI) scan. There was a no significant change in clinical status between cognitive assessment and MRI scanning. The cognitive domains of attention, verbal memory, executive function, and language were evaluated using the auditory continuous performance test (A-CPT), a verbal learning test, the Wisconsin Card Sorting Test (WCST), and a word-fluency test, respectively. Composite z-scores for each cognitive domain were calculated using the mean and standard deviation of HCs.

The scores for commission error and perseverative error were reversed so that better performance was indicated by positive z-scores. The domain composite scores constituted the average z-scores of (1) correct responses and commission errors for attention; (2) total recall (A1–A5), learning slope (A5–A1), and delayed recall at 20 min for verbal memory of 15 words; (3) categories completed and perseverative errors for executive function; and (4) tests on animals, stationery, ⌝, ⋏, and ° for language. Global cognitive function was calculated by averaging the z-scores of the four cognitive domains.

### MRI Scan Acquisition and Preprocessing

Three-dimensional T1-weighted images were obtained using magnetization-prepared rapid gradient echo (repetition time: 1,900 ms, echo time: 2.5 ms; flip angle: 9°; field of view: 250 mm^2^; image matrix: 256 × 246 mm; voxel size = 1.0 × 1.0 × 1.0 mm3; 176 slices) at Jeonbuk National University Hospital on a 3T Verio scanner (Siemens Magnetom Verio, Erlangen, Germany) using a 12-channel standard quadrature head coil.

Cortical surface reconstruction and volumetric segmentation of each subject's T1-weighted volumetric images were performed using FreeSurfer 6.0.0 (http://freesurfer.net/fswiki) (31). This resulted in a white matter and pial (i.e., gray matter) surface mesh for each subject. The Desikan–Killiany and aseg atlases were used for cortical and subcortical segmentation, respectively. Following visual quality checks (https://sites.bu.edu/cnrlab/lab-resources/freesurfer-quality-control-guide/), inaccuracies were manually edited using the voxel edit and recon edit tools in Freeview software and then corrected by reprocessing. Vertex-wise measures of cortical surface area (CSA) and CT as well as the CV, SCV, and intracranial volume (ICV) were estimated.

### Statistical Analyses

For demographic and clinical characteristics, we performed one-way ANOVA, *t*-tests, or Chi-square tests (as appropriate) using SPSS version 20.0 (SPSS, Inc.). Statistical significance was set at *p* < 0.05.

### Whole-Brain Analysis

Given potential effects of antipsychotics on brain structure (32, 33), we included CPZ equivalent as a covariate for CSA and CT, and CPZ equivalent and ICV as covariates for CV and SCV comparisons among the three groups. Patients with antipsychotic-naïve or -free state and all controls were given a chlorpromazine equivalent score of zero (34). All *p*-values in the ANCOVA and *post hoc* tests were corrected for multiple comparisons using a false discovery rate (FDR) correction threshold of *q* = 0.05. Only corrected results are presented. The percentage difference in CT between two groups was obtained using the formula [(thickness of group 1 (mm) – thickness of group 2 (mm)/thickness of group 2 (mm) × 100]. For CV and SCV, relative (%) volume [(absolute volume (cm^3^)/ICV (cm^3^) × 100] was calculated to control for subject head size, and the percentage difference between two groups was estimated using the same formula as for CT.

### Correlation Analysis

To assess the association between PANSS or cognitive function scores and structural measures of the brain regions where pairwise group differences were found, we performed partial correlation analyses with age, sex, and the CPZ equivalent as covariates for the TRS and ES-S groups. All *p*-values were corrected for multiple comparisons using the FDR correction threshold of *q* = 0.05.

### Subgroup Analysis

One study suggested that brain atrophy and CT in TRS may be contributed to by switching to clozapine treatment (6). Hence, to explore differential effects of clozapine vs. other atypical antipsychotics on structural measures in TRS, we divided participants into clozapine (*n* = 26) and no-clozapine (*n* = 20) groups depending on the type of medication at the time of scanning. Whole-brain analyses were repeated separately in the clozapine and no-clozapine groups. Comparison of clozapine and no-clozapine groups was also performed.

## Results

### Demographic and Clinical Characteristics and Cognitive Measures

No significant differences in age, sex, and education level were found among the TRS, ES-S, and HC groups. The TRS group exhibited a significantly younger age of onset, longer duration of illness (DI), greater CPZ equivalent dose, and higher negative PANSS subscores compared to the ES-S group. Twenty-two patients with ES-S were antipsychotic-naïve or -free at the time of the investigation. A comparison of the clozapine and no-clozapine groups is shown in Supplementary Table S6. Regarding the cognitive measures, both patient groups exhibited worse global cognitive function than did HCs. For the verbal memory domain, the composite score of the TRS group was significantly lower than that of the ES-S group (Table 1).

**Table 1:** Demographic and clinical characteristics and cognitive measures of patients with TRS and ES-S, and HC.

	**TRS (*n* = 46)**	**ES-S (*n* = 45)**	**HC (*n* = 61)**	**Statistics** (F/**χ^2^/t)**	**Significance (*p*-value)**	***post*−*hoc*^a^**
Age (years)	42.61 ± 9.90	38.04 ± 8.57	39.89 ± 9.52	*F* = 2.745	0.068	
Sex (M/F)	30/16	22/23	29/32	χ^2^ = 3.789	0.150	
Education (years)	13.73 ± 2.36	12.93 ± 3.03	13.33 ± 2.43	*F* = 1.222	0.297	
Age of onset (years)	24.17 ± 7.49	35.89 ± 9.20		*t* = 6.667	<0.001	
Duration of illness (years)	18.43 ± 9.30	1.72 ± 1.64		*t* = 12.005	<0.001	
CPZ equivalent dose (mg/d)	825.56 ± 398.45	234.62 ± 143.84 (*n* = 23)		*t* = 8.959	<0.001	
SOFAS	49.57 ± 9.36	54.93 ± 17.31		*t* = −1.835	0.071	
**PANSS**						
Positive subscore	16.76 ± 5.34	16.89 ± 8.07		*t* = −0.089	0.929	
Negative subscore	16.41 ± 7.08	13.22 ± 7.17		*t* = 2.026	0.046	
General subscore	28.78 ± 8.20	28.91 ± 9.51		*t* = −0.068	0.946	
Total score	61.96 ± 19.10	59.02 ± 21.43		*t* = 0.690	0.492	
**Attention**						
Correct response	114.76 ± 21.84	115.08 ± 18.35 (*n* = 41)	129.64 ± 6.31	*F* = 15.151	<0.001	3 > 1, 2
Commission error	13.38 ± 12.02 (*n* = 45)	13.17 ± 11.30 (*n* = 42)	5.03 ± 11.18	*F* =9 .261	<0.001	1,2 > 3
Composite score	−1.55 ± 1.14	−1.62 ± 1.26	0.00 ± 1.56	*F* = 15.590	<0.001	3 > 1, 2
**Verbal memory**						
Total recall (A1~A5)	34.46 ± 12.38	40.64 ± 12.89	49.69 ± 7.70	*F* = 26.406	<0.001	3 > 2 > 1
Learning slope (A5-A1)	4.07 ± 2.53	4.43 ± 2.12	5.70 ± 2.42	*F* = 7.147	<0.001	3 > 1, 2
Delayed recall (20 min)	5.48 ± 3.61	7.66 ± 4.07	10.34 ± 2.22	*F* = 29.315	<0.001	3 > 2 > 1
Composite score	−1.62 ± 0.82	−0.97 ± 0.39	0.00 ± 0.29	*F* = 29.785	<0.001	3 > 2 > 1
**Executive function**						
Category completed	3.32 ± 2.01 (*n* = 44)	3.93 ± 2.40 (*n* = 42)	5.31 ± 1.38	*F* = 15.151	<0.001	3 > 1, 2
Perseverative error	26.35 ± 15.28 (*n* = 43)	15.86 ± 12.16 (*n* = 37)	15.28 ± 11.51	*F* = 10.537	<0.001	1 > 2, 3
Composite score	−1.20 ± 0.34	−0.52 ± 0.67	0.00 ± 0.56	*F* = 13.314	<0.001	3 > 1, 2
**Language**						
Animals	14.09 ± 4.81	13.82 ± 3.84	19.57 ± 5.33	*F* = 25.152	<0.001	3 > 1, 2
Stationery	12.20 ± 5.15	13.30 ± 5.07	22.84 ± 6.40	*F* = 58.226	<0.001	3 > 1, 2
“⌝”	8.49 ± 3.99 (*n* = 45)	8.93 ± 4.11	13.49 ± 5.17	*F* = 20.190	<0.001	3 > 1, 2
“⋏”	7.96 ± 4.00 (*n* = 45)	8.57 ± 4.52	12.70 ± 4.07	*F* = 20.645	<0.001	3 > 1, 2
“°”	8.64 ± 4.30 (*n* = 45)	8.39 ± 4.28	12.38 ± 4.56	*F* = 13.942	<0.001	3 > 1, 2
Composite score	−1.13 ± 0.32	−1.07 ± 0.25	0.00 ± 0.33	*F* = 45.285	<0.001	3 > 1, 2
**Global cognitive function**	−1.37 ± 0.25	−1.05 ± 0.45	0.00 ± 0.72	*F* = 45.522	<0.001	3 > 1, 2

*For cognitive tests, unless otherwise specified, the sample sizes were 46, 44, and 61 for TRS and ES-S and HC, respectively*.

a *Bonferroni post-hoc test: 1, treatment-resistant schizophrenia; 2, early-stage schizophrenia; 3, healthy controls*.

*CPZ, Chlorpromazine; PANSS, Positive and Negative Syndrome Scale; SOFAS, Social and Occupational Functioning Assessment Scale; TRS, Treatment-Resistant Schizophrenia; ES-S, Early-Stage Schizophrenia; HC, Healthy Controls*.

### Whole-Brain Analysis

There were significant differences in CT among the three groups in the frontal (caudal and rostral middle frontal gyrus, lateral orbitofrontal cortex, pars opercularis, pars orbitalis, and pars triangularis), temporal (banks of the superior temporal sulcus, superior temporal gyrus, middle temporal gyrus, and inferior temporal gyrus), and parietal regions (inferior parietal cortex and supramarginal gyrus) and in the posterior cingulate cortex and insula. In the subsequent *post hoc* analyses, significantly greater reductions in the right pars opercularis (*t* = −3.617, *p* < 0.001), left pars triangularis (*t* = −3.331, *p* = 0.034), and left insula (*t* = −3.082, *p* = 0.045) were observed in the TRS group compared to the ES-S group. A comparison of TRS and HC groups revealed that all regions were significantly decreased in the TRS group. Significantly greater reductions in the right rostral middle frontal gyrus (*t* = −4.100, *p* < 0.001), right pars opercularis (*t* = −2.907, *p* = 0.045), right superior temporal gyrus (*t* = −3.096, *p* = 0.034), right inferior temporal gyrus (*t* = −3.472, *p* = 0.034), right inferior parietal cortex (*t* = −2.863, *p* = 0.049), and right supramarginal gyrus (*t* = −1.131, *p* = 0.034) were observed in the ES-S group compared to HC group (Table 2; Supplementary Figure S1).

**Table 2:** Comparison of cortical thickness among patients with TRS (*n* = 46) and ES-S (*n* = 45), and HC (*n* = 61).

**Structure**	**Hemisphere**	**TRS vs. ES-S vs.HC**		**TRS vs. ES-S**		**TRS vs.HC**		**ES-S vs. HC**	
		**F**	***p***	**% Difference**	***p***	**% Difference**	***p***	**% Difference**	***p***
Caudal middle frontal gyrus	Left	4.467	0.049	−1.99	0.401	−4.15	0.031	−2.21	0.105
Rostral middle frontal gyrus	Right	9.260	<0.001	−1.67	0.723	−5.10	0.020	−3.48	<0.001
Lateral orbitofrontal cortex	Left	5.214	0.027	−3.72	0.188	−5.64	0.011	−1.99	0.149
Pars opercularis	Right	13.141	<0.001	−3.37	<0.001	−5.55	<0.001	−2.25	0.045
Pars orbitalis	Right	4.610	0.044	−4.51	0.295	−7.40	0.020	−3.03	0.140
Pars triangularis	Left	7.838	0.010	−3.18	0.034	−4.20	<0.001	−1.05	0.229
	Right	7.840	0.010	−4.16	0.068	−6.08	<0.001	−2.00	0.121
Banks of superior temporal sulcus	Right	6.594	0.012	−3.40	0.130	−5.70	0.009	−2.38	0.112
Inferior temporal gyrus	Right	7.736	0.010	−1.13	0.468	−4.02	0.011	−2.92	0.034
Middle temporal gyrus	Left	6.057	0.016	−1.91	0.330	−4.21	0.016	−2.34	0.051
	Right	5.423	0.024	−2.51	0.313	−4.70	0.019	−2.25	0.082
Superior temporal gyrus	Left	7.314	0.010	−4.22	0.147	−6.73	<0.001	−2.62	0.076
	Right	9.780	<0.001	−3.88	0.091	−6.52	<0.001	−2.75	0.034
Inferior parietal cortex	Right	6.410	0.012	−2.08	0.313	−4.37	0.011	−2.34	0.049
Supramarginal gyrus	Right	6.247	0.012	−2.61	0.484	−5.44	0.020	−2.91	0.034
Posterior cingulate cortex	Right	6.720	0.012	−4.16	0.051	−5.54	<0.001	−1.44	0.229
Insula	Left	5.912	0.016	−4.57	0.045	−5.39	0.009	−0.86	0.460
	Right	4.975	0.034	−4.40	0.091	−5.51	0.011	−1.16	0.376

*TRS, Treatment-Resistant Schizophrenia; ES-S, Early-stage Schizophrenia; HC, Healthy Controls*.

*CPZ equivalent as covariate; p-value is a false discovery rate (FDR) corrected one*.

**Figure 1:**
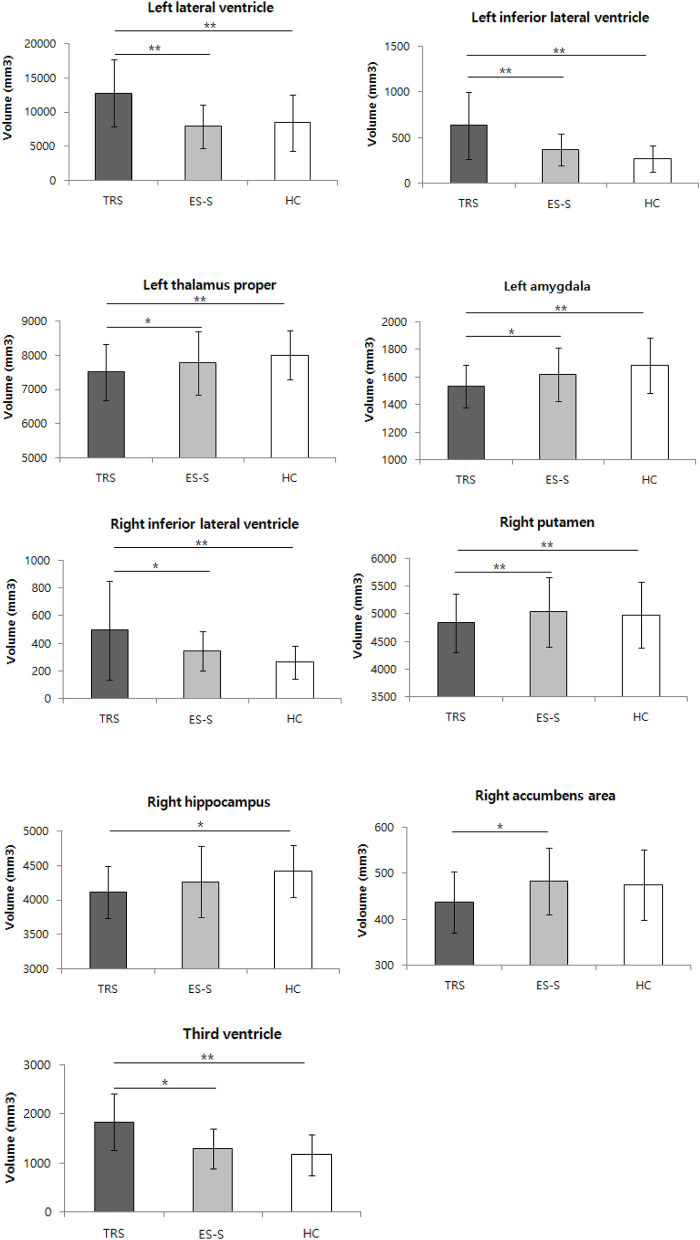
Comparison of subcortical volume among patients with treatment-resistant schizophrenia (TRS) and early-stage schizophrenia (ES-S), and healthy controls (HC). *p < 0.05; **p < 0.01 in *post-hoc* test.

The SCV differed significantly among the three groups in the accumbens area, amygdala, hippocampus, putamen, thalamus proper, lateral ventricle, inferior lateral ventricle, and third ventricle. In the *post hoc* analyses, compared to the ES-S group, significantly greater reductions in all regions with significant differences among the three groups except the hippocampus were observed in the TRS group [accumbens area (right: *t* = −2.928, *p* = 0.016), amygdala (left: *t* = −2.671, *p* = 0.024), putamen (right: *t* = −3.838, *p* < 0.001), thalamus proper (left: *t* = −3.015, *p* = 0.013), lateral ventricle (left: *t* = 4.061, *p* < 0.001), inferior lateral ventricle (left: *t* = 4.083, *p* < 0.001; right: *t* = 2.854, *p* = 0.018), and third ventricle (*t* = 3.062, *p* = 0.013)]. Compared to the HCs, significantly greater reductions in all regions that were significantly different among the three groups except the accumbens area were found in the TRS group [amygdala (left: *t* = −3.430, *p* = 0.004), hippocampus (right: *t* = −3.016, *p* = 0.010), putamen (right: *t* = −3.134, *p* = 0.007), thalamus proper (left: *t* = −3.648, *p* < 0.001), lateral ventricle (left: *t* = 3.619, *p* < 0.001), inferior lateral ventricle (left: *t* = 5.196, *P* < 0.001; right: *t* = 3.880, *p* < 0.001), and third ventricle (*t* = 3.725, *p* < 0.001)]. No significant differences were found between the ES-S group and HCs (Figure 1; Supplementary Table S1). The CSA and CV did not differ significantly among the three groups at the uncorrected level.

### Correlation Analysis

**Figure 2:**
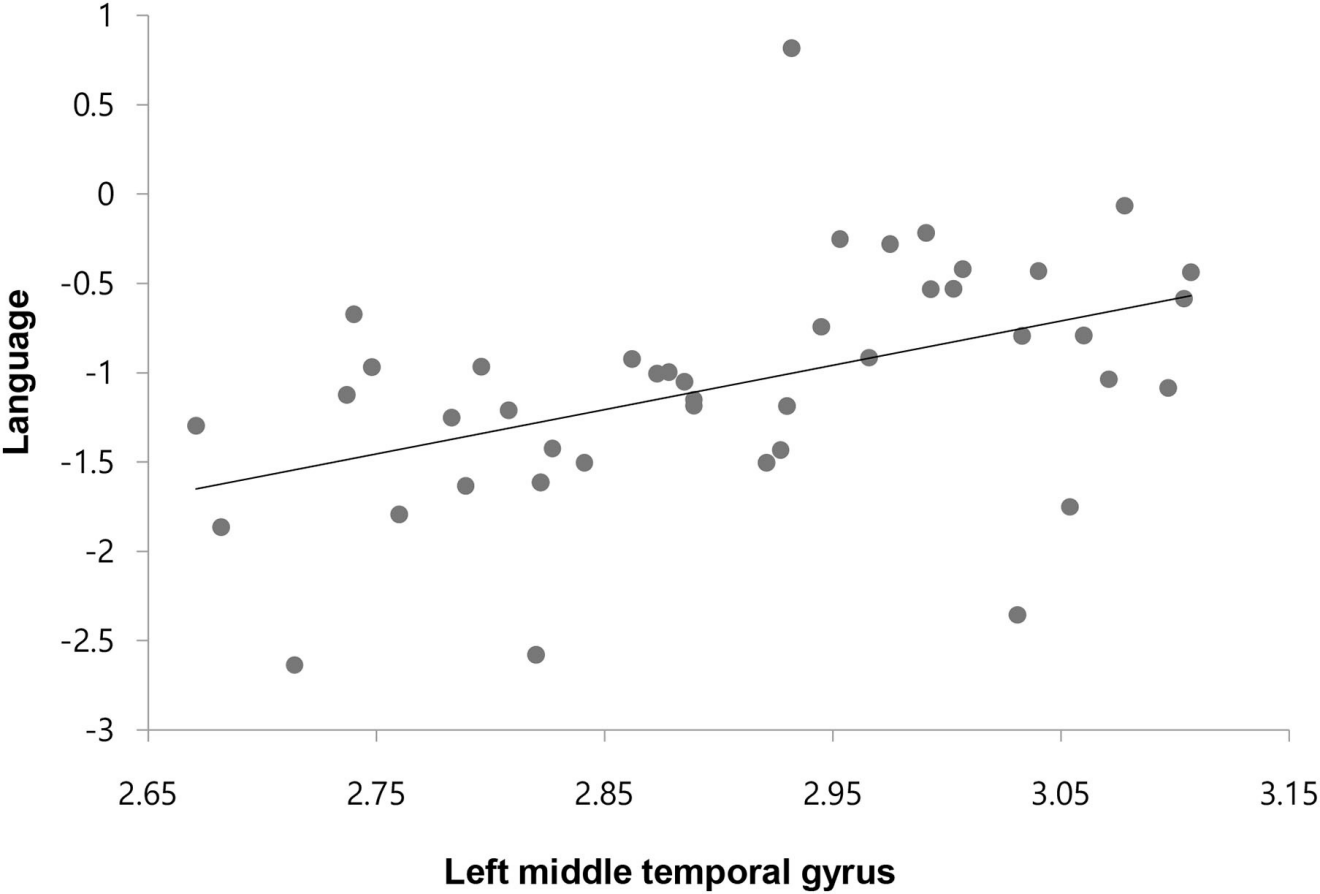
Correlation between the cortical thickness of left middle temporal gyrus and composite score of language in patients with early-stage schizophrenia.

We found a significant positive relationship only between the CT of the left middle temporal gyrus and the composite language score in the ES-S group with an FDR-adjusted *p*-value threshold (*r* = 0.516, *p* = 0.001) (Figure 2). For the results with an uncorrected *p*-value threshold, significant positive correlations were observed between cognitive function and CT of the orbitofrontal cortex, and temporal sulcus or pole whereas significant negative correlations of cognitive function were shown with the CT of the pericalcarine and cingulate cortices in the TRS group. For the PANSS scores, negative correlations were noted with the CT of the superior temporal gyrus but positive correlation was seen between the positive subscore and the CT of the entorhinal cortex (Supplementary Table S2). In the ES-S group, positive correlations of cognitive function were shown with the CT of the orbitofrontal and parietal cortices, pars opercularis, fusiform, precentral and temporal gyri, and superior temporal sulcus whereas negative correlations with the CT of the cingulate cortex. For the PANSS scores, negative correlations were noted with the CT of the frontal and supramarginal gyri and orbitofrontal cortex, but positive correlation was observed between the positive subscore and the CT of the superior temporal gyrus (Supplementary Table S3).

There were significant positive correlations between cognitive function and accumbens volume in the TRS group (Supplementary Table S4) whereas positive or negative correlations were observed between the PANSS scores and amygdala and hippocampus, and lateral ventricle, respectively in the ES-S group (Supplementary Table S5).

### Subgroup Analysis

When the clozapine TRS group was compared against ES-S and HC groups, almost the same results were obtained except additional significant differences in CT in the temporal region and differences in SCV in the corpus callosum. In the *post hoc* analyses, the results were roughly similar (for details, see Supplementary Tables S7, S8). In the analysis of the no-clozapine group, a significant difference was found only in the inferior lateral ventricle (Supplementary Table S9).

## Discussion

To investigate neuroanatomical abnormalities in TRS, values for CSA and CT as well as for CV and SCV were compared to those in the ES-S and HC groups. We observed a significant reduction in CT in the TRS group compared to the ES-S and HC groups and in the ES-S group compared to the HC group. For SCV, a significant reduction was found in the TRS group compared to the ES-S and HC groups. However, there was no difference between the ES-S and HC groups. Interestingly, the thickness of the left middle temporal gyrus in the ES-S group was significantly and positively correlated with scores in the language domain.

With respect to CT values, compared to HCs, our findings indicate that patients with TRS exhibited a widespread reduction in most cortical areas, including the frontal, temporal, and parietal cortices and the cingulate cortex and insula, consistent with the results of a previous study (5). On the other hand, a more recent study reported reduction of CT in more restricted regions, superior and inferior temporal gyri (4). Compared to the ES-S group, reductions were only noted in the inferior frontal gyrus and insula. This is in contrast to the results of two previous studies, which showed decreases in the left dorsolateral prefrontal cortex (5) and the temporal, parietal, occipital, and cingulate cortices (4). However, it should be noted that in those two studies, the comparison group comprised subjects with NTRS. With respect to CV, although no differences were found among the groups at an FDR-corrected *p*-value threshold, several regions (left precentral gyrus, right fusiform gyrus, right lateral orbitofrontal gyrus, right paracentral gyrus, right pars opercularis, right pars triangularis, and right superior frontal gyrus) were found to be reduced in the TRS group compared to the ES-S and HC groups with an uncorrected *p*-value threshold (data not shown). Other studies comparing TRS and HC groups have reported significant decreases in cortical volume in the middle and inferior temporal and lateral occipital gyri, insula, and precuneus (4) as well as in the frontal structures (8) in the TRS group. Furthermore, compared to the NTRS group, the TRS group has been found to have decreased volume in the frontal and precentral regions (4) as well as the superior and middle frontal gyri (8). Those two studies did not adjust for multiple comparisons. Notably, one study controlling for multiple comparisons reported reduced gray matter in the inferior temporal gyrus and central operculum compared to HCs and in the frontal, temporal, occipital, post-central, and supramarginal gyri compared to first-line antipsychotic responders (7).

As for SCV, we observed significant reductions in the amygdala, accumbens, putamen, thalamus, and hippocampus in the TRS group compared to the ES-S and HC groups, whereas significant increases were found in the lateral, third, and inferior lateral ventricles in the TRS group compared to the ES-S and HC groups. Previous studies also demonstrated that an ultra-resistant schizophrenia group exhibited a significantly smaller thalamic volume compared to HCs and smaller striatal and globus pallidus volumes compared to first-line antipsychotic responders (11). A smaller hippocampal volume was also found in TRS patients compared to HCs (10). However, conflicting results have been reported, ranging from no difference in SCV (13) to a larger corpus callosum in TRS subjects compared to controls (12). Two meta-analyses of longitudinal MRI studies concluded that in schizophrenia, there is progressive ventricular enlargement after illness onset greater than that seen in controls (35, 36). Importantly, our result showing greater ventricular enlargement in patients with TRS is the first such finding adopting modern criteria. Furthermore, the very large change, ranging from 40 to 137%, was surprising given that an average 16% enlargement of the lateral ventricle was previously reported in schizophrenia (37). This finding needs to be replicated in future studies.

What could be the underlying mechanisms for reduced CT and SCV in TRS in the present study? First, the reductions may be associated with the underlying neurobiology of TRS or the severity of illness. TRS, a more severe form of schizophrenia, is hypothesized to represent a separate and qualitatively different form of illness underpinned by a different neuropathological mechanism. The most prominent hypothesis is that TRS may reflect glutamate dysfunction despite normal dopamine regulation or even hypodopaminergic activity (38). Three magnetic resonance spectroscopy (MRS) studies have consistently demonstrated that glutamate levels in the anterior cingulate cortex (39, 40) or dorsolateral prefrontal cortex and putamen (41) were higher in patients with TRS compared with HCs or patients with schizophrenia who were treatment responsive. As elevations of glutamate have been associated with excitotoxicity and structural brain changes (42), glutamate increases in resistant patients could account for the relative gray matter reductions. To verify this hypothesis, longitudinal multimodal neuroimaging studies, i.e., MRI plus MRS and/or positron emission tomography, should be pursued. With regard to the effect of symptom severity on structural brain parameters, it can be speculated that more severe symptoms are associated with more severe pathophysiology in the brain, especially at the neurotransmitter level (glutamate, dopamine, or both), resulting in neuronal apoptosis and subsequent brain changes. However, evidence supporting this speculation is scarce. Instead, in a large longitudinal MRI study of patients with schizophrenia, disease severity had minimal or no effect on brain volume (43). Furthermore, as symptom severity measured by the PANSS did not differ between the two patient groups in the present study, this hypothesis seems unlikely.

Second, the reductions in CT and SCV may be associated with the potential neurotoxicity of antipsychotics. Two meta-analyses reported that patients with schizophrenia consistently exhibited a significantly greater loss of total cortical gray matter volume over time, and this was related to cumulative antipsychotic intake during the interval between scans (32, 44). Moreover, long-term administration of antipsychotics in monkeys (45) and rats (46) also resulted in significant decreases in brain volume. Antipsychotic-associated neuronal changes in the brain are altered expression levels of proteins affecting cell survival, impairment of the mitochondrial respiratory chain, increases in DNA fragmentation, injury to dendritic microtubules, increases in dopamine-generated reactive oxygen species, changes in cell morphology, and rapid induction of apoptosis (47). This explanation may be supported by the greater difference in the CPZ-equivalent dose between the two patient groups and weaker or no differences in CT/SCV between the ES-S and HC groups relative to those between the TRS and HC groups. As 26 subjects in the TRS group were taking clozapine, it is worth considering the effects of clozapine with regard to the current findings. The relationship between clozapine treatment in TRS and changes in brain functioning is as yet unclear, and the results have been inconsistent. A review of 23 relevant articles revealed that the use of clozapine was associated with volume reduction in the basal ganglia, especially the caudate nucleus, where functional neuroimaging studies also found decreased perfusion. In the frontal lobe, clozapine treatment was associated with increased or decreased gray matter volume and perfusion (48). To delineate separate effects of clozapine, we conducted the entire set of analyses again with clozapine and no-clozapine groups. The results for the clozapine group were similar to those for the whole group, whereas in the no-clozapine group, we found a significant difference only in the inferior lateral ventricle. This may indicate that the clozapine group was the primary contributor to findings for the whole TRS group. A possible explanation might be that psychopathology in the clozapine group was more severe before switching to clozapine. These severe symptoms before clozapine may have caused structural abnormalities. However, symptom severity was decreased and became no different than no-clozapine group because of therapeutic effects of clozapine. Or it may be related to detrimental effects of clozapine given that increased cortical thinning was associated with clozapine (43, 49) and increased pro-apoptotic caspase-3 was observed in rat frontal cortex following treatment with clozapine (50). Nevertheless, it should be pointed out that only few regions (CT of the cuneus, CV of the banks of superior temporal sulcus, SCV of the corpus callosum) were significantly different between clozapine and no-clozapine groups (Supplementary Tables S10–S14) suggesting that degree of structural abnormalities in the no-clozapine group is between clozapine and ES-S groups. Regardless of underlying mechanisms for reduced CT and SCV in the TRS, an important implication of the findings may be that inferior frontal gyrus and insula are crucial brain regions in terms of preventing disease progression from early-stage to chronic resistant stage. The functions of inferior frontal gyrus are related to efficiency of semantic processing (51), controlled retrieval of lexical representations (52), semantic fluency (53), and integrating meanings of word to sentence-level (54). The insula is involved in sensorimotor processing, empathy, social cognition and salience processing (55). Interestingly, one study found that the left anterior insular gray matter volume was greater in first episode psychosis group compared to chronic psychosis (56). Therefore, it may be inferred that therapeutic interventions minimizing or recovering pathological changes in these regions are helpful.

We observed significant cognitive impairment in both the TRS and ES-S groups compared to the HC group. In the comparison of TRS and ES-S subjects, only verbal memory was significantly worse in the TRS group, similar to another study (17). However, most previous studies reported overall cognitive dysfunction in TRS compared to NTRS (20, 57, 58). This may be due to the mild level of symptoms in our TRS group (mean PANSS total score, 61.96 ± 19.10), whereas other studies recruited TRS patients with mean PANSS total scores ranging from 71 to 91. To understand the relationship between cognitive impairment and altered brain structure in TRS, correlation analysis was performed. Unexpectedly, no significant results were found with an FDR-adjusted *p*-value threshold. However, it should be noted that at the uncorrected *p*-value, we observed significant positive or negative correlations of the CT and SCV of multiple brain areas with cognitive function in the TRS and ES-S groups (Supplementary Tables S2–S5). Furthermore, a significant positive correlation between language and the thickness of the middle temporal gyrus at the FDR corrected threshold was found in the ES-S group. This suggests that a structural abnormality in the middle temporal gyrus may contribute to the deficit in language function in the ES-S group. Taken together, these findings imply that association of cognitive dysfunction with structural changes is relatively evident in the early-stage of schizophrenia but may disappear over deteriorating progression to TRS. It may be that different mechanisms other than altered brain structures such as long DI, chronic exposure to antipsychotics and social deprivation, are contributing to cognitive dysfunction in TRS. For correlation results between the CT and PANSS scores, it was of interest to see a positive correlation of the CT in the superior temporal gyrus with the positive subscore in the ES-S group but a negative correlation of the CT in the same region with positive, general and total scores in the TRS group. It may be interpreted that initial hyperdopaminergic state and subsequent brain atrophy due to neuroinflammation or antioxidant damages are responsible for these changes.

Our study's limitations are as follows. First, we did not recruit more specific subtypes of TRS as recommended by the Treatment Response and Resistance in Psychosis Working Group (2). The proportions of the positive, negative, and positive and negative subtypes were 21.7%, 8.7%, and 41.3%, respectively. The remaining 28.3% did not meet the criteria for the positive or negative domain because we applied a rating of moderate severity on just one symptom item. This issue should be considered in designing future studies. Second, 19.2% of the clozapine group did not meet the criteria for positive or negative specifiers. Therefore, those patients could be classified as clozapine responsive. The heterogeneity of our TRS patients limits the interpretation of the results, especially with regard to the role of clozapine. Third, because of limited sample size, the results of subgroup analysis with clozapine/no-clozapine groups should be interpreted cautiously. Fourth, the CPZ-equivalent dose and DI in the ES-S group differed from those in the TRS group. To disentangle the confounding effects of the dosage and exposure duration of antipsychotics and DI, the selection of comparator groups that are carefully matched regarding these factors is necessary. Lastly, the time difference between MRI scan and cognitive tests may have masked possible correlations between structural changes and cognitive dysfunction. The strengths of our study are that a) we measured four structural brain parameters (CSA, CT, CV, and SCV) as well as cognitive function in patients with TRS and b) we used two age-, sex-, and education-matched comparator groups, i.e., the ES-S and HC groups.

## Conclusion

In the current study, our findings indicate that structural changes and cognitive impairment were greater in the TRS group compared to the ES-S and HC groups. The association of structural abnormalities with cognitive dysfunction was evident in the early-stage but absent in the resistant stage. To prevent disease progression, inferior frontal gyrus and insula may be important target areas.

## Data Availability Statement

The original contributions presented in the study are included in the article/Supplementary Material, further inquiries can be directed to the corresponding author.

## Ethics Statement

All patients were recruited at Jeonbuk National University Hospital and volunteered to participate in the study; all provided written informed consent. This study has been approved by the Ethics Committee of Jeonbuk National University Hospital (approval number: CUH 2012-08-001). The patients/participants provided their written informed consent to participate in this study.

## Author Contributions

Y-CC conceptualized the study. W-SK, JS, UT, and N-IK performed the study and acquired data. CL and W-SK conducted statistical analysis. CL drafted the manuscript. N-IK, K-HL, JS, and W-SK critically reviewed the manuscript and Y-CC finalized it. All authors approved the final manuscript.

## Funding

This study was supported by a grant from the Korean Mental Health Technology R&D Project, Ministry of Health and Welfare, Republic of Korea (HL19C0015) and a grant from the Korea Health Technology R&D Project through the Korea Health Industry Development Institute (KHIDI) funded by the Ministry of Health and Welfare, Republic of Korea (HI18C2383).

## Conflict of Interest

The authors declare that the research was conducted in the absence of any commercial or financial relationships that could be construed as a potential conflict of interest.

## Publisher's Note

All claims expressed in this article are solely those of the authors and do not necessarily represent those of their affiliated organizations, or those of the publisher, the editors and the reviewers. Any product that may be evaluated in this article, or claim that may be made by its manufacturer, is not guaranteed or endorsed by the publisher.
