# Supplementary material for: Altered Neuroanatomical Signatures of Patients With Treatment-Resistant Schizophrenia Compared to Patients With Early-Stage Schizophrenia and Healthy Controls
Source: Front Psychiatry. 2022 May 18;13:802025. doi: 10.3389/fpsyt.2022.802025 (PMC9158464; doi:10.3389/fpsyt.2022.802025)
Supplement: Supplementary file 1 [file Data_Sheet_1.pdf]

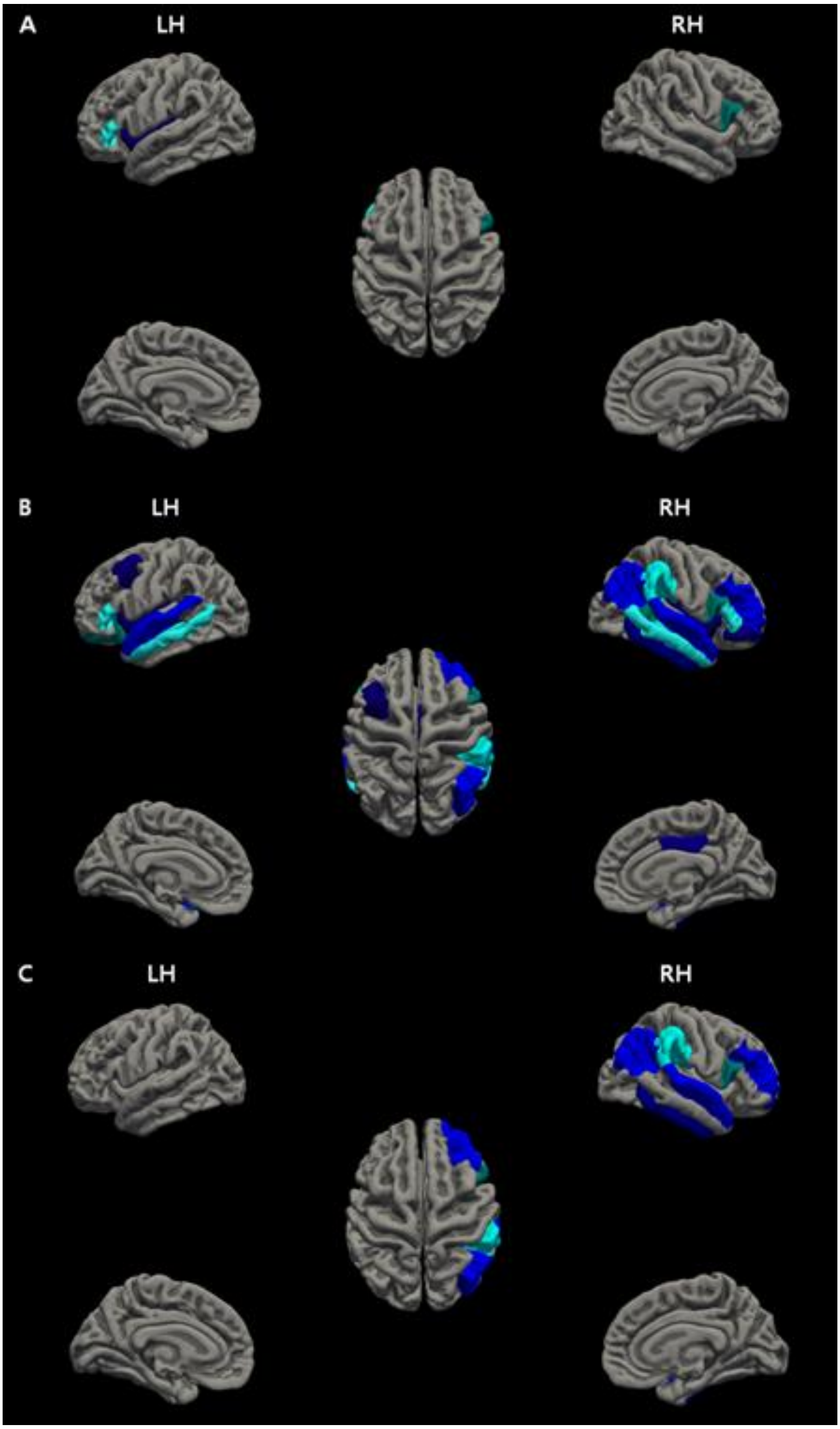

Figure S1. Brain regions exhibiting differences in cortical thickness A) between the TRS and ES-S groups, B) between the TRS and HC groups and C) between the ES-S and HC groups.
Results are shown at $p < 0.05$ corrected for multiple comparisons with false discovery rate (FDR). Regions colored in blue indicate significantly decreased cortical thickness compared to the ES-S group or HC group. LH, left hemisphere; RH, right hemisphere.

Table S1. Comparison of subcortical volume among patients with TRS (n=46) and ES-S (n=45), and HC (n=61)

Structure	Hemisphere	TRS vs. ES-S vs.HC		TRS vs. ES-S		TRS vs.HC		ES-S vs. HC	
		F	p	% Difference	p	% Difference	p	% Difference	p
Amygdala	Left	5.915	0.012	-7.40	0.024	-9.50	0.004	-2.27	0.502
Accumbens area	Right	4.383	0.042	-11.44	0.016	-8.81	0.080	2.97	0.841
Hippocampus	Right	4.737	0.033	-5.42	0.091	-7.54	0.010	-2.24	0.502
Putamen	Right	7.366	0.005	-5.90	<0.001	-3.41	0.007	2.64	0.841
Thalamus proper	Left	6.656	0.009	-5.45	0.013	-7.01	<0.001	-1.65	0.566
Lateral ventricle	Left	8.367	<0.001	56.15	<0.001	50.62	<0.001	-3.54	0.883
Inferior lateral ventricle	Left	13.553	<0.001	68.35	<0.001	136.60	<0.001	40.54	0.502
	Right	7.702	0.005	43.22	0.018	92.40	<0.001	34.34	0.502
Third ventricle		6.936	0.005	40.15	0.013	57.22	<0.001	12.18	0.566

Abbreviation: TRS, Treatment-Resistant Schizophrenia; ES-S, Early-Stage Schizophrenia; HC, Healthy Controls.

ICV and CPZ equivalent as covariates; P value is a false discovery rate (FDR) corrected one. The % difference was calculated from the relative volume.

Table S2. Correlations between the cortical thickness and PANSS and cognitive function in patients with treatment-resistant schizophrenia

Structure	Hemisphere	PANSS and cognitive function	Treatment-resistant schizophrenia	
			r	p[a]
Lateral orbitofrontal cortex	Left	Verbal memory	0.304	0.048
	Right	Executive function	0.311	0.048
		Global cognitive function	0.363	0.017
Medial orbitofrontal cortex	Right	Global cognitive function	0.315	0.039
Banks of superior temporal sulcus	Right	Attention	0.335	0.028
Superior temporal gyrus	Right	Positive subscore	-0.316	0.039
		General subscore	-0.306	0.046
		Total subscore	-0.303	0.048
Temporal pole	Left	Negative subscore	-0.311	0.042
	Right	Attention	0.364	0.016
		Global cognitive function	0.363	0.017
Entorhinal cortex	Left	Positive subscore	0.455	0.002
Pericalcarine cortex	Right	Verbal memory	-0.381	0.012
		Attention	-0.364	0.016
		Global cognitive function	-0.345	0.024
Posterior cingulate cortex	Right	Verbal memory	-0.328	0.032

[a]Uncorrected p value

Table S3. Correlations between the cortical thickness and PANSS and cognitive function in patients with early-stage schizophrenia

Structure	Hemisphere	PANSS and cognitive function	Early-stage schizophrenia	
			r	p[a]
Caudal middle frontal gyrus	Right	Positive subscore	-0.322	0.037
Lateral orbitofrontal cortex	Left	Language	0.340	0.029
	Right	Negative subscore	-0.317	0.041
		Language	0.320	0.041
Pars opercularis	Left	Verbal memory	0.346	0.027
		Language	0.439	0.004
	Right	Global cognitive function	0.351	0.025
Pars orbitalis	Right	Attention	-0.360	0.024
		Language	0.314	0.046
Precentral gyrus	Left	Verbal memory	0.370	0.017
		Global cognitive function	0.366	0.019
Banks of superior temporal sulcus	Right	Global cognitive function	0.344	0.028
Inferior temporal gyrus	Left	Language	0.423	0.006
Middle temporal gyrus	Left	Language	0.516	0.001
	Right	Executive function	0.370	0.020
		Language	0.405	0.009
		Global cognitive function	0.314	0.045
Superior temporal gyrus	Left	Positive subscore	0.322	0.038
Fusiform gyrus	Left	Global cognitive function	0.314	0.046
Inferior parietal cortex	Right	Language	0.311	0.048
Superior parietal cortex	Right	Global cognitive function	0.336	0.032
Supramarginal gyrus	Right	General subscore	-0.403	0.008
		Total subscore	-0.333	0.031
		Verbal memory	0.397	0.010
		Global cognitive function	0.326	0.037
Posterior cingulate cortex	Right	Attention	-0.354	0.027

	Executive function	-0.318	0.048
	Global cognitive function	-0.331	0.035

[a]Uncorrected p value

Table S4. Correlations between the subcortical volume and PANSS and cognitive function in patients with treatment-resistant schizophrenia

Structure	Hemisphere	PANSS and cognitive function	Treatment-resistant schizophrenia	
			r	p[a]
Accumbens area	Right	Verbal memory	0.393	0.009
		Language	0.366	0.016
		Global cognitive function	0.384	0.011

[a]Uncorrected p value

Table S5. Correlations between the subcortical volume and PANSS and cognitive function in patients with early-stage schizophrenia

Structure	Hemisphere	PANSS and cognitive function	Early-stage schizophrenia	
			r	p[a]
Amygdala	Left	General subscore	0.336	0.029
Hippocampus	Right	Positive subscore	0.309	0.047
		General subscore	0.353	0.022
Lateral ventricle	Left	General subscore	-0.354	0.021

[a]Uncorrected p value

Table S6. Demographic and clinical characteristics of clozapine and no-clozapine groups

	Clozapine (n=26)	No-clozapine (n=20)	t-test	p value
Age (years)	42.88 ± 9.88	42.25 ± 10.17	t = 0.213	0.832
Sex (M/F)	17/9	13/7	χ^2 = 0.001	0.978
Education	13.75 ± 2.46	13.70 ± 2.27	t = 0.071	0.944
Age of onset (years)	23.62 ± 8.18	24.90 ± 6.62	t = -0.572	0.570
Duration of illness (years)	19.48 ± 9.62	17.06 ± 8.91	t = 0.873	0.387
CPZ equivalent dose (mg/d)	716.83 ± 403.17	966.91 ± 353.47	t = -2.198	0.033
SOFAS	49.23 ± 8.57	50.00 ± 10.51	t = -0.273	0.786
PANSS				
Positive subscore	17.50 ± 5.81	15.80 ± 4.63	t = 1.072	0.290
Negative subscore	17.08 ± 7.39	15.55 ± 8.49	t = 0.615	0.518
General subscore	28.96 ± 7.28	28.55 ± 10.06	t = 0.161	0.873
Total score	63.54 ± 17.18	59.90 ± 21.63	t = 0.636	0.528

Abbreviation: TRS, Treatment-Resistant Schizophrenia; PANSS, Positive and Negative Syndrome Scale; SOFAS, Social and Occupational Functioning Assessment Scale. Means and standard deviations are reported.

Table S7. Comparison of cortical thickness among patients with clozapine group of TRS (n=26) and ES-S (n=45), and HC (n=61)

Structure	Hemisphere	TRS vs. ES-S vs.HC		TRS vs. ES-S		TRS vs.HC		ES-S vs. HC	
		F	p	% Difference	p	% Difference	p	% Difference	p
Caudal middle frontal gyrus	Right	5.678	0.025	-3.49	0.911	-3.81	0.117	-0.33	0.017
Rostral middle frontal gyrus	Right	8.859	<0.001	-0.88	0.820	-4.33	0.044	-3.48	<0.001
Lateral orbitofrontal cortex	Left	5.788	0.025	-4.11	0.109	-6.02	0.010	-1.99	0.136
Pars opercularis	Right	9.129	<0.001	-3.77	0.091	-5.94	<0.001	-2.25	0.060
Pars triangularis	Left	6.859	0.011	-3.86	0.068	-4.87	<0.001	-1.05	0.253
	Right	4.807	0.040	-3.97	0.188	-5.89	0.019	-2.00	0.136
Banks of superior temporal sulcus	Right	6.586	0.017	-4.09	0.109	-6.37	0.010	-2.38	0.104
Inferior temporal gyrus	Right	8.441	<0.001	-1.73	0.301	-4.59	0.010	-2.92	0.017
Middle temporal gyrus	Left	5.053	0.039	-1.58	0.457	-3.88	0.042	-2.34	0.060
	Right	4.707	0.042	-2.92	0.334	-5.10	0.032	-2.25	0.089
Superior temporal gyrus	Left	7.443	0.011	-4.06	0.109	-6.57	<0.001	-2.62	0.060
	Right	8.687	<0.001	-4.37	0.109	-7.00	<0.001	-2.75	0.041
Temporal pole	Left	5.027	0.039	-4.78	0.068	-3.97	0.024	0.85	0.952
Inferior parietal cortex	Left	5.229	0.039	-2.10	0.153	-3.59	0.014	-1.53	0.136
	Right	5.967	0.023	-2.04	0.334	-4.33	0.021	-2.34	0.057
Supramarginal gyrus	Right	6.813	0.017	-1.80	0.436	-4.66	0.021	-2.91	0.017
Posterior cingulate cortex	Right	4.863	0.040	-3.89	0.109	-5.28	0.014	-1.44	0.246
Insula	Right	4.771	0.040	-4.84	0.109	-5.94	0.014	-1.16	0.374

Abbreviation: TRS, Treatment-Resistant Schizophrenia; ES-S, Early-Stage Schizophrenia; HC, Healthy Controls.
CPZ equivalent as covariate; p value is a false discovery rate (FDR) corrected one.

Table S8. Comparison of subcortical volume among patients with clozapine group of TRS (n=26) and ES-S (n=45), and HC (n=61)

Structure	Hemisphere	TRS vs. ES-S vs.HC		TRS vs. ES-S		TRS vs.HC		ES-S vs. HC	
		F	p	% Difference	p	% Difference	p	% Difference	p
Amygdala	Left	5.202	0.030	-6.74	0.039	-8.86	0.008	-2.27	0.463
Accumbens area	Right	4.352	0.049	-11.96	0.024	-9.35	0.078	2.97	0.806
Thalamus proper	Left	4.692	0.039	-6.57	0.035	-8.11	0.011	-1.65	0.717
Putamen	Right	7.751	0.010	-7.88	0.000	-5.45	0.008	2.64	0.806
Lateral ventricle	Left	8.295	<0.001	70.54	<0.001	64.50	0.006	-3.54	0.865
Inferior lateral ventricle	Left	12.076	<0.001	90.37	<0.001	167.55	<0.001	40.54	0.462
	Right	6.329	0.016	61.92	0.035	117.52	0.006	34.34	0.462
Third ventricle		4.962	0.031	44.12	0.035	61.68	0.008	12.18	0.671
CC central		5.690	0.020	-9.92	0.011	-10.27	0.006	-0.39	0.806

Abbreviation: TRS, Treatment-Resistant Schizophrenia; ES-S, Early-Stage Schizophrenia; HC, Healthy Controls; CC, Corpus Callosum.
ICV and CPZ equivalent as covariates; p value is a false discovery rate (FDR) corrected one. The % difference was calculated from the relative volume.

Table S9. Comparison of subcortical volume among patients with no-clozapine group of TRS (n=20) and ES-S (n=45), and HC (n=61)

Structure	Hemisphere	TRS vs. ES-S vs.HC		TRS vs. ES-S		TRS vs.HC		ES-S vs. HC	
		F	p	% Difference	p	% Difference	p	% Difference	p
Inferior lateral ventricle	Left	8.307	<0.001	39.72	0.171	96.36	<0.001	40.54	0.031
	Right	8.485	<0.001	18.91	0.171	59.74	0.016	34.34	0.031

Abbreviation: TRS, Treatment-Resistant Schizophrenia; ES-S, Early-Stage Schizophrenia; HC, Healthy Controls.
ICV and CPZ equivalent as covariates; p value is a false discovery rate (FDR) corrected one. The % difference was calculated from the relative volume.

Table S10. Comparison of cortical thickness between patients with clozapine group of TRS (n=26) and no-clozapine group of TRS (n=20)

Structure	Hemisphere	Clozapine vs. No-clozapine	
		% Difference	p
Cuneus	Right	3.54	0.030

Abbreviation: TRS, Treatment-Resistant Schizophrenia.
p value is a false discovery rate (FDR) corrected one.

Table S11. Comparison of subcortical volume between patients with clozapine group of TRS (n=26) and no-clozapine group of TRS (n=20)

Structure	Hemisphere	Clozapine vs. No-clozapine	
		% Difference	p
CC Central		-17.94	0.018
CC middle anterior		-16.04	0.023

Abbreviation: TRS, Treatment-Resistant Schizophrenia; CC, Corpus Callosum.
p value is a false discovery rate (FDR) corrected one.

Table S12. Comparison of subcortical volume between patients with clozapine group of TRS (n=26) and no-clozapine group of TRS (n=20)

Structure	Hemisphere	Clozapine vs. No-clozapine	
		% Difference	p
CC middle anterior		-16.04	0.045

Abbreviation: TRS, Treatment-Resistant Schizophrenia; CC, Corpus Callosum.
CPZ equivalent, age of onset and duration of illness as covariate; p value is a false discovery rat e (FDR) corrected one.

Table S13. Comparison of cortical volume between patients with clozapine group of TRS (n=26) and no-clozapine group of TRS (n=20)

Structure	Hemisphere	Clozapine vs. No-clozapine	
		% Difference	p
Banks of superior temporal sulcus	Right	-10.24	0.023

Abbreviation: TRS, Treatment-Resistant Schizophrenia; p value is a false discovery rate (FDR) corrected one.

Table S14. Comparison of cortical volume between patients with clozapine group of TRS (n=26) and no-clozapine group of TRS (n=20)

Structure	Hemisphere	Clozapine vs. No-clozapine	
		% Difference	p
Banks of superior temporal sulcus	Right	-10.24	0.035

Abbreviation: TRS, Treatment-Resistant Schizophrenia.
CPZ equivalent, age of onset and duration of illness as covariate; p value is a false discovery rat e (FDR) corrected one.
